# Reflections of physiotherapy students in the United Arab Emirates during their clinical placements: A qualitative study

**DOI:** 10.1186/1472-6920-5-3

**Published:** 2005-01-20

**Authors:** Hélène Larin, Jean Wessel, Amal Al-Shamlan

**Affiliations:** 1 Chair, Department of Physiotherapy, College of Health Sciences, University of Sharjah, Sharjah, United Arab Emirates, a project in affiliation with McMaster University, Hamilton, ON, Canada; 2 Professor, School of Rehabilitation Science, McMaster University, Hamilton, ON, Canada, L8S 2L4; 3 Lecturer, Department of Physiotherapy, University of Sharjah, Sharjah, United Arab Emirates

## Abstract

**Background:**

Although Western models of education are being used to establish health professional programs in non-Western countries, little is known about how students in these countries perceive their learning experiences. The purpose of this qualitative study was to describe the reflections of physiotherapy students from a Middle East culture during their clinical placements and to compare them to reflections of physiotherapy students from a Western culture.

**Methods:**

Subjects were six senior students (3 females, 3 males, mean age 22.6 years) and 15 junior, female students (mean age 20.1 years) in the baccalaureate physiotherapy program at a university in the United Arab Emirates (UAE). They wrote weekly entries in a journal while in their clinical placements. They described an event, their reaction to it, and how it might affect their future behavior. Two evaluators independently read and coded the content of all the journals, and then worked together to categorize the data and develop themes. A third evaluator, an UAE national, independently read the journals to validate the content analysis. A feedback session with students was used to further validate the data interpretation. The themes were compared to those derived from a similar study of Canadian physiotherapy students.

**Results:**

The content of the students' reflections were grouped into 4 themes: professional behavior, awareness of learning, self-development and shift to a patient orientation, and identification and analysis of ethical issues. Although the events were different, students from the UAE considered many of the same issues reflected on by Canadian students.

**Conclusion:**

Physiotherapy students from a Middle East culture consider many of the same issues as students from a Western culture when asked to reflect on their clinical experience. They reflect on their personal growth, on how they learn in a clinical setting, and on the ethical and professional behaviors of themselves and others.

## Background

Reflection is considered by many [1-3] in the Western world to be an important part of clinical practice and clinical decision-making. In order to learn from experience, health care professionals must reflect on the events and determine where the information 'fits' in relation to their assumptions, previous experience and knowledge. Reflection is the process where individuals think about and evaluate their experience in order to come to new understandings and appreciations [2-4]. All promoters of reflection refer to it as a process of thinking about experiences. The process leads to a change in perspective or understanding. Schön [2,3] suggested that reflective practitioners are better able to manage the uncertainty and complexity of clinical practice, and therefore asserted that a major part of preparation of professionals should be centred on enhancing their ability to reflect.

Journal writing is a method that has been used to promote reflective thinking in health science students and professionals [5,6], to facilitate the link between academic learning and clinical practice [7], and to assist students to explore and change their attitudes toward patients [7,8]. When writing a reflective journal, students are asked to describe a learning event or situation, explain how the experience led to new understandings and appreciations, and consider how they might act differently in the future. Students have indicated that the writing of the journal provides a framework for the reflective process).)[9,10]. Shepard and Jensen [11] specifically referred to the need for physiotherapists to have 'reflective' knowledge in order to deal with uncertainties and value conflicts.

Two recent Canadian studies [12,13]. have described the reflections of physiotherapy students during clinical placement. The students wrote weekly in journals, and reflected on a number of topics that were classified into six major themes [12]. These included: 1) the process of making clinical decisions; 2) the complexity and richness of interactions with clients; 3) the influence of the practice environment on learning and patient care; 4) acquisition of clinical and administrative skills; 5) the value of clinical experiences in validating and integrating previous learning; and 6) acknowledgment and evaluation of different learning methods. Students frequently commented on their own values and beliefs and whether these were in harmony or conflict with the beliefs and actions of other health professionals or clients [13]. They discussed professional behavior, professional collegiality, respect for others, advocacy and informed consent.

In considering the student reflections, the investigators wondered about the potential impact of culture and working environment on the content of the reflective journals. Would students from a non-Western culture and a different health care system be concerned about the same issues? Would they feel comfortable with the reflective process, particularly documenting their thoughts in a journal? How might the hospital/institutional infrastructure and norms affect their learning?

We had a unique opportunity to answer some of these questions by comparing the themes from reflective journals of physiotherapy students in the United Arab Emirates (UAE) with those taken from the above mentioned studies [12,13]. conducted at McMaster University in Canada. The physiotherapy program at the University of Sharjah in the UAE was developed with a philosophy similar to that of the program at McMaster, and the Chair of the program was a McMaster faculty member. Students in both programs have worked in small groups, studied similar content, and considered the same professional and attitudinal issues such as moral reasoning, client-centred care, professional behavior and professional code of ethics.

Although both the UAE and Canada have many of the conveniences of modern society, there are major societal and cultural differences. The students in both countries have similar standards of living and have ready access to education and health services. Many of the health problems of modern society – diabetes, heart disease, arthritis, cancer, and trauma from traffic accidents – are common in Canada and the UAE. However, Canada is a democracy with a predominantly Christian and European population, whereas the UAE is a system of emirates with a population comprising mainly Arabic and Eastern cultures and where the Muslim religion predominates. The students in the two countries might be expected to have different values and beliefs, and different societal norms that could affect their reflections.

The purpose of this study was to describe the reflections of physiotherapy students from the University of Sharjah during their clinical placements. A second objective was to compare the themes generated from the Sharjah data to the themes from the previous two studies on reflections of McMaster students during their clinical placements. Because the students in the two programs have similar socioeconomic status and similar curricula, it was anticipated that differences in reflections would be due to differences in culture and life experiences related to the cultures.

## Methods

This qualitative study was conducted during the students'clinical placements in a summer semester of the Physiotherapy Program at the University of Sharjah, United Arab Emirates, a program developed in affiliation with McMaster University, Hamilton, Ontario, Canada.

### Curriculum and semester description

The baccalaureate physiotherapy program in the UAE comprises one pre-professional year (year 1) and three professional years (years 2–4) of study. To be admitted to the program, students must have completed the UAE Secondary School Scientific Certificate (equivalent to grade 12), or equivalent program accredited by the Ministry of Education. They must have also scored at least 500 on the Test of English as a Foreign Language (TOEFL).

While in the physiotherapy program, students need to complete university required and elective courses. Basic science courses are offered in years 1 and 2. Physiotherapy courses in years 2–4 include small-group, problem-based learning tutorials and clinical skills laboratories based on problems/ case scenarios enhanced with resource sessions. The male and female students are taught separately in discrete areas of the campus.

### Students

Six senior physiotherapy students (3 females and 3 males) with a mean age of 22.6 years (range 20.5 to 23.1) and 15 junior, female students with a mean age of 20.1 years (range 19.9 to 22.8) formed the subject group of this study. These students were respectively from the first and second cohorts of students admitted to the new physiotherapy program at the University of Sharjah. All students spoke Arabic and English. The senior students had completed the academic component of year 3 and were in their third clinical placement, which provided them with six weeks experience primarily with clients with cardio-respiratory conditions. The junior students had just completed two academic semesters of year 2, and were in their first clinical placement – four weeks with clients with peripheral musculoskeletal conditions. Students practiced in five different clinical sites in the UAE, under the supervision of clinical preceptors and university instructors/coordinators. The study was approved by the Ethics Committee of the College of Health Sciences, and all participating students signed an informed consent.

### Evaluators

Two instructors were involved in reading, coding and identifying the major themes that emerged from the students' reflective journals. One instructor (HL) from the University of Sharjah, Department of Physiotherapy, had taught one course to the junior group and two courses to the senior group. The second instructor (JW) from McMaster University Physiotherapy Program, had taught one course to the senior group as a consultant to the program. The two instructors were not involved with the students during their summer clinical placements. A third evaluator (AAS) independently read all the journals. She was an instructor in the Sharjah program, a UAE national, and fluent in both Arabic and English. She had taught two courses to the junior students. Her analysis was used to confirm the interpretation of the entries made by the other two evaluators who were both from Western cultures.

### Procedure

The students were asked to write a reflective journal, a new exercise for all students. This assignment was one of the requirements for a Pass mark in their summer clinical placement. The students were required to make one entry per week for the duration of their clinical placement (four weeks for junior students and six weeks for senior students), and to submit the journal weekly to the university instructor involved in their clinical supervision. However, the instructor did not provide any feedback to the student at that time. The students were instructed to include the following in their reflective journals:

*Reflective journals should include observations, impressions, and reactions to what you have learned in the academic portion of the semester and how you are applying it to clinical practice. How does the clinical experience change what you thought, felt, or did in the past, and how you may respond in the future? You are expected to write at least one journal entry per week during your clinical placement*.

1. Describe the learning event, issue or situation. Describe prior knowledge, feelings or attitudes with new knowledge, feelings or attitudes.

What happened?

2. Analyse the learning event, issue or situation in relation to prior knowledge, feelings or attitudes.

What was your reaction to the learning event, issue or situation? Your response may include cognitive and emotional reactions. Why did it happen?

3. Verify the learning event, issue or situation in relation to prior knowledge, feelings or attitudes.

What is the value of the learning event, issue or situation that has occurred? Is the new knowledge, feeling or attitude about the learning event, issue or situation correct?

4. Gain a new understanding of the learning event, issue or situation.

What is your new understanding of the learning event, issue or situation?

5. Indicate how the new learning event, issue or situation will affect future behavior. Determine the clarification of an issue, the development of a skill or the resolution of a problem.

How will you approach the same or similar event, issue or situation in the future?

### Data analysis

The method described by Coffey and Atkinson [14] was used to analyse and interpret the data in the students' journals. The analysis was conducted in two phases after the students had completed their clinical placements and submitted their entire journal. In Phase 1, one evaluator (HL) selected five journal entries based on their readability level and diverse content and forwarded them to the second evaluator (JW). The two evaluators read and coded the five journals independently. Then they met to discuss and establish agreement on the coding. In Phase 2, the two evaluators independently read and coded all remaining journals. They met again to 1) determine agreement on the content of each entry; 2) group the codes into categories; and 3) determine major themes. When coding discrepancy occurred, the evaluators discussed the discrepancy and came to a consensus.

The following two methods were used to further validate the analysis and interpretation of the data. 1) The third evaluator independently read and labelled all journal entries to confirm the interpretation of the content. 2) The themes established by the evaluators were presented to two students who had completed the journals, and who volunteered to provide feedback on the authors' interpretation of their reflections.

Themes for the present study were developed without direct reference to the themes found in the Canadian studies [12,13]. The Emirati and Canadian themes were then compared by examining their descriptions and content.

## Results

The two initial evaluators (HL, JW) came to a consensus on four themes concerning the clinical learning experience of the students. These themes did not change as a result of the coding of the third reader (AAS) or the feedback from the students. However, these additional data occasionally resulted in a change in emphasis of some of the content within a theme. Whenever this happened, the differences are described.

All students outlined the positive impact and value of their learning from the clinical placement. They discussed events and issues that fit into the following four themes:

1. Professional behaviors: skills and attitudes, scope of practice, time management, professional boundaries, and respect for clients and colleagues.

2. Awareness of learning: clinical versus academic learning, gaining of important new knowledge, self-directed and life-long learning, and client as a source of learning.

3. Self-development and shift to a more client-oriented focus over the time of the clinical placement.

4. Identification and analysis of ethical issues.

Each of these themes is discussed below with quotes provided for illustrative purposes.

### Professional behaviors

The students appeared very aware of their own behavior and that of other health professionals and students. They reflected on how they should behave as professionals, but this also included how they should behave in general. The events that precipitated discussion on this theme included actions of themselves and other health professionals and included what they regarded as both positive and negative behaviors. They referred to physiotherapists and other health professionals who helped the students and who modelled good professional behavior. They also described some situations where health professionals did not show sufficient respect for their clients or their colleagues, or did not provide 'best practice'. They commented on professional boundaries, being concerned about the client-therapist relationship becoming too personal and limits to the scope of physiotherapy practice. In addition, the students noted that self-directed learning and life-long learning were professional responsibilities. This topic is discussed under the next theme, Awareness of Learning.

The students providing feedback emphasized that professional behavior (both negative and positive) was also learned from patients who might show their appreciation, be patient or uncooperative, or provide specific feedback regarding the student's communication or skills. The students also provided examples of "crossing boundaries" in terms of professional competencies and relationships with their patients and supervising therapists.

*One patient asked me angrily where [were the physiotherapists]? I responded politely to wait until they finish their work with the patients... I calmed her down and asked her to be patient...I really felt that the [physiotherapist] and I should maintain and manage our time...It is really a big responsibility we have*. [student 11, week 3]

*The child came crying and refusing to look at her forearm. We should respect the emotional status of the child and try to understand it because the child has been through a lot. The accident..., surgery,...and functional limitations...The stress should be controlled before giving physiotherapy...I should be patient and try to give the most enjoyable set of exercises to gain the child's trust*. [student 10, week 3]

*Sometimes patients are shy to tell that there is no benefit from the treatment programme. So I asked the patient if he feels any improvements and if not to inform me in order to change the plan of treatment. The patient responded very well to that*. [student 5, week 3]

### Awareness of learning

Although the entire reflective journal assignment was designed to have students reflect on their learning, this theme was included to capture the many components of learning discussed by the students. They gained knowledge, but also commented on the unique aspects of clinical learning. Clinical experiences provided an opportunity to apply and enhance their academic learning. Junior students particularly noted how observing surgery helped them understand their anatomy, or how working with "real patients' was not as straight forward as their academic learning. The senior students realized the need to be life-long and self-directed learners because they were always encountering new conditions/situations and they wanted to provide their clients with 'best practice'. Students acknowledged the clients as a source of knowledge, and noted that clinical situations provided a stimulus to learn.

In the feedback session, students particularly emphasized how the clinical experience had enhanced their learning "a lot!" They also indicated that clinicians reinforced the need for life-long learning because of the constant changes in science. Some therapists welcomed new knowledge from students, for example, learning about outcome measures, while others indicated they did not have the time.

*It was really good to have a mix of people [clinicians] and to learn from their experience in the work field...Each one of them has given us a new way to deal with patients and... also a new experience to add to our future knowledge... The learning is not finished...We have to keep our hands on the best information available and the best experience*. [student 4, week 6]

*[I] try to apply the theoretical bases learned at the university. [I] interact with patients and take their histories, try to know their goals and what they need from physiotherapy. I try to invent new materials for the exercises. In my opinion, when we apply what we learned in the theory, the information sticks in our minds and is hardly forgotten*. [student 21, week 2]

### Self-development and orientation shift

The theme of self-development was derived from the content of individual student entries, but was also seen in the change in the reflections as students proceeded through their placements. Often at the beginning of the clinical encounter, the students were unsure of themselves, but they gained confidence as they worked with the client and/or learned from their colleagues. The change across the journal entries was even more noticeable. In many of the early entries, students described their own emotional response to a client or a situation. They were concerned about their own performance, and about what they were learning from the placement. They indicated how an event had an impact on them. The later entries were more client-centred. The focus was more on how the client responded to an event, or how helpful a physiotherapist was. Students also referred to an increased confidence, the more experiences they had.

It became evident from the third reader's summary that students were aware of how their professional behavior, outside direct patient care, could ultimately have an impact on the patient. They referred specifically to the maintenance of knowledge and skills and to relationships with other health professionals.

Students in the feedback session referred to their initial lack of confidence which improved during the clinical placement. They added, however, that it was difficult to demonstrate the "correct confidence level" to their instructor/evaluator, particularly when students were occasionally told they were "over-confident". They felt that self-development was affected by behavior of other physiotherapy students, method of feedback from instructors and whether their clients improved or not.

The quote immediately below illustrates how a student confronted his/her uncertainties during one client encounter. The next quote concerns a student's acknowledgement of the change in his/her confidence with increasing experience.

*First patient with tetraplegia...What if I do something wrong?...First, worried... and unable to do anything, I observed other colleagues... I could do it too! *[student 6, week 2]

*A one-month old infant was in the ICU. I thought I would not be able to touch her as usual, but things are now different. I did all the treatment... with my hands with no hesitation and I was not scared... My reaction was totally changed and it is becoming more regular... I am happy... I trust myself better and it is increasing with time...and will increase the more cases I face*. [student 3, week 6]

In the quotes below, the student initially talks about his/her own emotional response to a patient, but later in the placement discusses the need to adapt to patients' situations and provide them with the necessary treatment. In both journal entries, the student discusses a client with a novel and somewhat "unpleasant" condition. However, the student reacts differently in week 5 than week 1.

*At that moment I was shocked because I was not familiar with the neurology cases. When the physiotherapist asked me to help...I hesitated in the beginning. ...This situation had a bad effect on me ... I was depressed and I couldn't do any single movement *[student 4, week 1]

*I have seen a patient who was always under sedation due to his pain and his anger which was there almost all the time. ... I was always confused ...not knowing what to do for him and what is the best for him. ... Such a case made me think [very] much before any and each simple movement. ... So this made me prevent [other] problems such as ulcers, and invent any simple movement just to change his lying position *[student 4, week 5]

As the quotes illustrate, the student was concerned about what to do in both cases, but by week 5 was able to overcome the emotional response, problem solve about the management of the client, and take action.

### Ethics

Students commented on what they believed to be ethical or unethical behavior of the health professionals they were in contact with. They noted when informed consent was not obtained from clients and they discussed ways to get consent from uncooperative clients. They talked about having respect for their clients, and maintaining confidentiality. They noted "ethical" and "unethical" behavior in the same professionals under different circumstances.

*The staff is cooperative and helpful, respect each other, but there is no confidentiality or respect of time with patients*. [student 20, week 1]

They were concerned when individuals (clients or professionals) were being treated unfairly or differently because of their health condition or socio-economic status. In some cases they thought that the client might be harmed by the practice. One student was concerned about how to 'tell the truth' to a client who had a poor surgical result possibly due to the questionable practice of the surgeon.

One student described an event where a therapist refused to see a patient when she changed her appointment time, but treated another patient who did not have an appointment. The student felt that the latter patient was treated because she was a "VIP (very important person)".

*I felt sad for the [person who did not receive treatment], and I said to myself, where are the ethics? I learned before ... that all patients should be at the same level without any bias. ...all persons who work in health care fields specially the professional therapists must [follow] the code of ethics because they have humans in their hands. ... I will delete the word VIP from my life work*. [student 12, week 2]

In the feedback session, the students also indicated how clients could be discriminated against because of their health condition and the therapist's level of confidence. Therapists might provide less treatment to a child if they felt uncomfortable treating children. They might leave a student to treat an older person if they felt the management of older persons was less important or less effective.

### Feedback from students

The students agreed with the four themes and did not think that any major concept was omitted in this analysis. However, they added some insight to the content and interpretation of each theme. The students emphasized that many people affect their learning – university instructors, therapists, other health professionals, students and patients/clients. They felt that the information derived from the analysis of the reflective journals was very important. They strongly recommended that the results be disseminated to clinicians, instructors and future students to increase their awareness of the learning process and help the students to improve.

### Comparisons of themes of students from McMaster University and the University of Sharjah

The content of the journals indicated that students from both universities were comfortable with the reflective process. They frequently shared quite personal information, including their positive and negative emotions, concerns about their own behavior, and a discussion of their own beliefs. Some students in both countries, simply described an event and provided minimal personal reflection, but these students were in the minority.

Table 1 summarizes the comparable themes of the Emirati and Canadian students. Themes from both countries related to professional behaviors, learning and ethical issues. Self-development was not identified per se as a theme in the Canadian studies, but change and growth were mentioned under the other themes.

**Table 1:** Themes from Emirati and Canadian students during clinical placements

UAE	Canada
Professional behaviors	† Process of making clinical decisions
	† Complexity and richness of interactions with patients
	‡ Respect: for uniqueness of individual
	‡ Professionalism: responsibility and behavior as a member of a profession
	‡ Professional collegiality: interaction with and respect for health professionals including physical therapists

Awareness of learning	† Effects of practice environment on learning and patient care
	† Acknowledgment and evaluation of different learning methods
	† Value of clinical experiences in validating and integrating previous learning
	† Acquisition of knowledge and clinical and administrative skills

Identification and analysis of ethical issues	‡ Allocation of resources ‡ Advocacy: for clients, society or health policy ‡ Informed consent: right of person to decide what will happen to him/her

† Themes from Williams et al [12]

‡ Themes from Geddes et al [13]

In the area of professional behavior, students in both countries commented on role models (both positive and negative) in the clinical environment, as well as on their own behavior or response to clients. Both groups demonstrated a respect for their clients and saw their role as going beyond physical treatment. Canadian students mentioned advocacy in the community while students in the UAE discussed supporting their clients within the hospital milieu.

Both groups talked about the uniqueness of learning in clinical placements. The clinical experience enhanced and confirmed their academic learning, and stimulated them to learn more. They enjoyed learning new information and using their knowledge to help others. They were anxious when they "didn't know what to do", but expressed increased confidence with each experience. They appreciated constructive feedback on their performance from clinical supervisors, health professionals and clients. Both groups acquired new skills related to direct client care (therapeutic techniques and communication) and administrative activities (time management, organizational skills and charting). Only Canadian students referred to writing reports and billing, tasks that students in the UAE were not expected to do in their clinical placements. Both groups, however, broadened their view of the scope of practice of physiotherapy.

Students from both countries noted ethical issues pertaining to inequalities in their respective health care systems. Canadian students were concerned about the shortfall of resources (time, personnel, funding), and about differences in health care provided to private clients or those funded through provincial health insurance. Emirati students noted that the health or societal status of a client could affect their treatment within the health system.

## Discussion

Physiotherapy students from Sharjah demonstrated the ability to reflect on their clinical practice at the highest level (discussion of how experience will impact on future behavior). They described not only the learning event, but also their emotional and cognitive reactions to it. They analysed the situations to arrive at new understandings and considerations of how they would behave differently in the future.

Like the students in the Canadian studies, the subjects in the present study described three types of learning that are considered essential for clinical decision-making. These are propositional (academic learning – facts, concepts), professional craft (learning from experience), and personal knowledge (knowledge of self and unique frame of reference) [15]. The students commented on the knowledge that they gained (e.g., anatomy, treatment techniques, medications), and the unique learning opportunities of fieldwork (e.g., application and enhancement of academic learning, learning new skills, response to novel situations). They acknowledged that their personal frame of reference affected their clinical interactions, but was also modified by their clinical experience. Although the students did not categorize their learning in the three areas, they were able to describe the acquisition of knowledge and skills required for making clinical decisions.

The reflection on one's own development is a common theme in the reflections of health professionals. Jensen and Denton[7] referred to themes of student self-adequacy and inadequacy in their study of 23 physiotherapy students completing journals in their first clinical placement. Tryssenaar and Perkins[16] demonstrated that reflection on personal growth was still evident at the end of the educational programs and the beginning of careers for physical and occupational therapists. Subjects in their study described initial anxiety and then increasing confidence about their ability as graduate therapists. They became more patient-centred with experience.

Jensen and Paschal[17] suggest that the shift to a client-centred approach is part of the transition that students go through from their first to later clinical placements. Because we did not follow one group of students over several placements, we cannot comment on the change in the focus of students with each additional clinical experience. However, both junior and senior students described their personal reactions more frequently at the beginning of the placement, and the patient's unique situation more frequently in the later weeks. Perhaps with each new experience (e.g., start of a clinical placement) students will be temporarily more self-centred as they deal with their anxieties and uncertainties. As they become more comfortable with their own abilities, they can focus more on the client. Such a pattern is in agreement with the finding mentioned above, that therapists become more client-centred with experience. [16]

One other study [18] examined the reflections of non-Western physiotherapy students during their clinical placements. Although the students were practicing in an English, Western environment, rather than in their own culture and language, the students and their supervisors did raise some issues that may have relevance to the present study. The Chinese students and their Australian supervisors noted that the students were reluctant to self-evaluate, to express their opinions, to admit they did not understand or to take active steps to acquire needed information. In the present study, the students certainly voiced their opinions, admitted their perceived inadequacies, and expressed pride in their achievements in the reflective journals. However, some may have been less forthcoming with their clinical preceptors or university instructors/coordinators, particularly when they did not agree with them. In the sample quotes in the Results section, the students did not mention discussing their concerns and feelings with their supervisors. On the other hand, the students in the present study were working in their own language and culture, and would be better able to express their views, and to do it in a "politically correct" manner. Unlike the Chinese students, the students in the UAE discussed the need for self-direction in their learning. This difference could be cultural and/or due to the self-directed nature of the problem-based learning method used in the physiotherapy program at the University of Sharjah.

Language might have been a factor in the reflections of the students from the UAE, however. Although the students worked in Arabic in the clinical environment, they conducted their physiotherapy education and wrote their journals in English. It is possible that some students had difficulty accurately expressing their reflections in English, and that there was some misinterpretation by the evaluators. However, two of the evaluators (HL, AAS) knew the students well and were used to their writing. One of them (AAS) was a UAE national and fluent in both English and Arabic. When the two initial evaluators (HL, JW) worked together in Phase 1 to determine coding methods, part of the discussion was on how to interpret the "second-language writing" of the journals. In spite of these precautions, it is still possible that the themes may have varied somewhat if the journals had been written in Arabic and interpreted by Arabic-speaking evaluators.

## Conclusions

Physiotherapy students from a Middle East culture consider many of the same issues as students from a Western culture when asked to reflect on their clinical experience. They reflect on their personal growth, on how they learn in a clinical setting, and on the ethical and professional behaviors of themselves and others.

## Competing interests

The author(s) declare that they have no competing interests.

## Authors' contributions

HL and JW were involved in the conception and design of the study, in the qualitative analysis, and in the writing of the manuscript. HL coordinated the data collection. AAS assisted with data analysis. All authors were involved in the preparation of the manuscript, and read and approved the final version.

## Pre-publication history

The pre-publication history for this paper can be accessed here:


